# Lunar rhythms and their carry-over effects may shape environmental sex determination in a coral reef fish

**DOI:** 10.1098/rspb.2024.0613

**Published:** 2024-08-07

**Authors:** Jeffrey S. Shima, Suzanne H. Alonzo, Craig W. Osenberg, Erik G. Noonburg, Stephen E. Swearer

**Affiliations:** ^1^ School of Biological Sciences, Victoria University of Wellington, Wellington, New Zealand; ^2^ Department of Ecology and Evolutionary Biology, University of California at Santa Cruz, Santa Cruz, CA, USA; ^3^ Odum School of Ecology, University of Georgia, Athens, Georgia, USA; ^4^ 7001 Seaview Avenue NW, Suite 160 PMB 860, Seattle, WA, USA; ^5^ Oceans Institute, University of Western Australia, Crawley, Australia

**Keywords:** alternative mating strategies, developmental plasticity, lunar cycle, post-settlement processes, protogynous hermaphrodite, reproductive tactic

## Abstract

Lunar rhythms shape spawning phenology and subsequent risks and rewards for early life-history stages in the sea. Here, we consider a perplexing spawning phenology of the sixbar wrasse (*Thalassoma hardwicke*), in which parents spawn disproportionately around the new moon, despite the low survival of these larvae. Because primary sex determination in this system is highly plastic and sensitive to social environments experienced early in development, we ask whether this puzzling pattern of spawning is explained by fitness trade-offs associated with primary sexual maturation. We used otoliths from 871 fish to explore how spawning on different phases of the moon shapes the environments and phenotypes of settling larvae. Offspring that were born at the new moon were more likely to settle (i) before other larvae, (ii) at a larger body size, (iii) at an older age, (iv) to the best quality sites, and (v) as part of a social group—all increasing the likelihood of primary maturation to male. Selection of birthdates across life stage transitions suggests that the perplexing spawning phenology of adults may reflect an evolutionarily stable strategy that includes new moon spawning for compensatory benefits later in life, including preferential production of primary males at certain times.

## 1. Introduction

Biological sex has fundamental consequences for fitness [1,2] and is environmentally determined during early development for many animals [3,4]. Nest temperature, [5,6], photoperiod [7] and social environment [8–11] often shape sexual differentiation (i.e. maturation as the male or female phenotype) in reptiles, amphipods and fishes, respectively. Parents may influence the developmental conditions of their offspring (and by extension, sex determination) by choosing where or when to reproduce [12]. Such parental effects can work in concert with developmental plasticity [13] to shape reproductive strategies that maximize fitness, particularly when environmental variation can be accurately predicted by parents. However, the extent to which parents exploit the lunar cycle to manipulate the sex of their offspring remains largely unexplored.

This may be an oversight because the lunar cycle drives substantial environmental variation in the sea, and it is entirely predictable. In addition to its well known effects on tides [14], the moon shapes the movements of a community of pelagic organisms that migrate vertically in the water column on a daily cycle [15–18]. In particular, the timing and intensity of moonlight regulate nightly influxes of both vertically migrating prey and predators for the pelagic larvae of reef fishes [19], to create predictable variation in risks and rewards in the ocean's surface waters on a 29.5 day cycle. Consequently, reef fishes are known to capitalize on this predictable variation [20–22], and are expected to use this information to maximize survival and fitness of their offspring.

Fishes in the genus *Thalassoma* generally exhibit lunar periodicity in spawning [20–26], and notable plasticity in their mating systems [27,28] and developmental patterns [8,29]. Species within this genus are generally considered protogynous hermaphrodites, with most individuals maturing first to females [30,31]. Individuals can change sex later in life, and this is regulated by size-based agonistic social interactions [9,32,33] that alter hormone pathways to regulate sex change [30,34–36]. Females generally incur lower risks of mortality relative to males, and their mating opportunities are all but assured. However, changing sex can greatly increase an individual's fitness in certain conditions [37]. Males trade-off mortality risk for increased mating opportunities (i.e. with many females), and sex-change in *Thalassoma* is well described by the size-advantage model [38,39].

Alternative reproductive tactics are another important feature of this system [2,27,39]. Most individuals will reproduce first as females, and socially dominant females may subsequently undergo a sex change to become males (hereafter, secondary males). However, some individuals mature directly as males (hereafter, primary males). These two male types are phenotypically distinct. Secondary males have a ‘terminal phase’ phenotype: they are larger, socially dominant, and morphologically distinct (i.e. differing in body shape and ornamentation) from ‘initial phase’ females and males. Terminal phase males—which comprise both secondary males and primary males that survive to attain this phenotype [40]—attempt to monopolize matings through defence of spawning sites or harems of females. By contrast, younger (and smaller) primary males mimic female phenotypes and behaviour patterns, and this facilitates their surreptitious matings within territories of terminal phase males [27,28,35]. Primary males are also adapted for sperm competition. Their higher sperm production rates give them a fitness advantage over terminal phase males when spawning involves a group of individuals [41–44], and this strategy is favoured at higher population densities [28].

Importantly, initial sex differentiation (i.e. from juveniles to either females or primary males) in this group is environmentally sensitive and determined early in ontogeny [8]. At lower population densities, primary males are rare; at higher population densities, primary males are proportionally more common [28,39]. An elegant experiment by Munday *et al*. [8] confirms that the social context early in development drives this pattern: in the absence of social interactions (i.e. at low population densities), juveniles mature mainly as females. When juveniles are reared in groups (i.e. at higher population densities), typically one fish within each group (usually the largest) matures as a primary male [8].

Our previous work on the sixbar wrasse, *Thalassoma hardwicke*, has documented lunar rhythms that shape spawning patterns [22,25], larval growth and survival [22,29], and phenotypes of juveniles [12,22]. Adults reproduced most often around the new moon even though this was associated with lower survival of their offspring. This paradox led us to assume the existence of a compensatory benefit later in an offspring's life that favours this parental spawning pattern. Such compensatory effects could include differential survival in post-settlement life stages, and/or patterns of primary or secondary sexual maturation that may be associated with offspring spawned at a new moon. To identify the stage(s) and demographic processes that could compensate for the observed mismatch, we address the following questions: (1) Are the traits of settling larvae that were born during the new moon likely to confer post-settlement fitness benefits? (2) Do the spawning patterns of parents lead to different sex determination patterns for their offspring (e.g. the likelihood of being a female, primary male, or terminal phase male)? (3) Can selection operating across successive life stages explain the spawning patterns of adults? Answers to these questions will help to solve an ecological puzzle for *Thalassoma*, and more broadly, will highlight a potentially novel form of environmental sex determination driven by parental spawning strategies and the lunar cycle.

## 2. Methods

### (a) Study system and sample collection

The sixbar wrasse (*Thalassoma hardwicke*) is a small-bodied reef fish, and a common resident of shallow lagoon habitats on Mo'orea, French Polynesia. Adult sixbars spawn pelagic larvae that develop at sea for approximately 46 days, (range: 37–61 days; [22]). Larval sixbars settle around the new moon, to small patch reefs [45], either alone or in small groups [46], and undergo metamorphosis within branching corals or macroalgae [47]. Larvae vary in their developmental histories [29], and consequently, settlers vary in size and age [12,22]. Recently settled fish compete primarily with other similarly aged individuals for suitable habitat [48]. These agonistic interactions are further mediated by a settler's relative timing of arrival to the reef (i.e. priority effects; [49,50]), site quality [51,52], local density [46,51], and group size [46,53]. Work conducted on another species of *Thalassoma* [8] suggests that any of these traits of young individuals and their rearing environments can influence initial sex differentiation.

As part of an earlier study, we quantified spawning and patterns of larval development of sixbars from Mo'orea, French Polynesia [22]. Specifically, we estimated birthdates and larval durations of sixbars that survived to successive developmental stages (i.e. settlement, older juvenile, adult), and identified a striking pattern of lunar periodicity in larval growth [29]. Here, we use a sample of otoliths from 871 fish (411 settlers, 291 juveniles and 169 adults; see [22] for details of fish collections) to evaluate the association between adult spawning and the post-settlement traits and reproductive outcomes for their offspring. The sample of adults comprised: 61 females, 62 primary males, and 46 terminal phase males. All fish were collected under protocols approved by Victoria University of Wellington's Animal Ethics Committee (permit numbers: 22038 and 26378), and with the permission of the Delegation à la Recherche (de la Polynésie Française).

### (b) Relationships between spawning patterns and offspring traits

To partially evaluate the hypothesis that compensatory benefits later in an offspring's life might explain the puzzling pattern of parental spawning at the new moon, we evaluated rearing environments and phenotypes of all recently settled fish in the sample (*n* = 411 settlers). To facilitate later comparisons with adults (see below), we binned birthdates of individuals to their nearest lunar quarter of birth (i.e. new moon, waxing (synonymous with first quarter) moon, full moon and waning (synonymous with third or last quarter) moon), using the lunar.phase function of the R package ‘lunar’ [54].

We evaluated trait distributions of settlers as a function of their lunar quarter of birth. More specifically, we visualized distributions of (1) the timing of settlement relative to the new moon (i.e. a proxy for a priority effect in which fish that settled prior to the new moon had a competitive advantage); (2) otolith radius at settlement (a proxy for body size); (3) the number of daily otolith increments between hatch and settlement check marks (i.e. a proxy for age at settlement); (4) settlement to offshore versus inshore sites (i.e. a proxy for site quality: offshore sites are of higher quality than inshore sites; [51,52]); and (5) settlement to reefs in groups or alone (i.e. a proxy for social context, e.g. [8]). This final trait was estimated from a subset of samples for which we also had data on local population size (*n* = 141 individuals). Based on prior studies of this species (cited above), and prior research on a congener [8], we assumed that fish had an advantage (in terms of survival, and the probability they would mature into primary males) if they settled before the new moon, at a large size, at an older age, and to offshore reefs.

### (c) Trait-based reconstructions of adult birthdates

Birthdates of young fish can be estimated directly from daily growth increments and a known date of capture [22]. Larval growth patterns of adult fish can also be reliably obtained from otoliths; however, the large number of daily otolith increments after settlement precludes reliable direct estimation of an adult's age (and hence, their birthdate). Thus, we used a set of otolith-based traits to infer birthdates of adults to the nearest lunar quarter.

We know from our prior work that fish age and birthdate (i.e. lunar period) affect larval growth. Thus, to quantify growth patterns that allowed us to infer birthdates, we first removed age-related trends in growth rates of sixbar wrasse across their larval stage [29]. Briefly, we (1) evaluated daily otolith increments from 871 fish, (2) estimated otolith radius at each age (*R_i_*), (3) fitted a linear model (using 'lm' in R [55]: *R_i_*∼‘age’, with age modelled as a factor), (4) obtained residuals for each observation as a measure of de-trended size-at-age, (5) interpreted a change in residual size (modified by Eqn (2.4) in [29] to account for settlement-related effects) as a measure of ‘residual growth’. These residual growth patterns differ for fish born during different lunar periods owing to effects of moonlight [29]. For some subsequent steps (identified below) we normalized (i.e. centred and scaled) these residuals, and smoothed each time series (i.e. residual growth across the larval stage for a given fish) using a 3 day rolling average.

**Figure 2.**
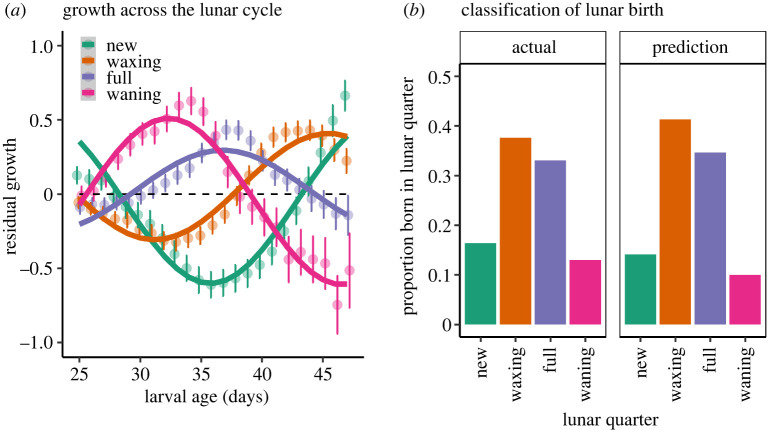
(*a*) Patterns of residual larval growth across the lunar cycle vary with lunar quarter of birth for the sixbar wrasse. Residual (i.e. age-independent) growth is mean ± 1 s.e., fitted by periodic regression. (*b*) Validation of birthdate classification: actual distribution of birthdates for settlers and juveniles corresponds closely to predicted birthdates for settlers and juveniles, accomplished by linear discriminant analysis (with leave-one-out cross-validation, overall classification success = 50.9%).

We estimated a set of 12 traits related to the cyclical pattern of larval growth driven by moonlight [29] (see also figure 2*a*), and used these traits in a linear discriminant analysis ('lda', MASS package [56]) to assign birthdates to the nearest lunar quarter. First, we used the 'tsfeatures' package [57] to estimate three traits (trend’, ‘linearity’ and ‘curvature) of each time series of residual growth (normalized and smoothed) across the complete larval stage. These three traits were estimated using the stl_features method, whereby ‘trend’ is estimated by STL decomposition; ‘linearity’ and ‘curvature’ were calculated from coefficients of an orthogonal quadratic regression. Second, for each individual fish, we fitted linear models of residual growth (normalized and smoothed) to larval age (using ‘lmList’), obtaining slopes and intercepts for three targeted age ranges (28–34, 35–41 and 42–48 days post-hatch), which yielded six traits. Third, we constrained each time series to 25–47 days post-hatch to fit a periodic function to residual growth (in this case, not normalized and not smoothed, to preserve the inherent variability within each time series):

2.1 Residual growth=a×sin⁡((θ×π/180)+c)+d,

where *a, c* and *d* were estimated separately for each individual, using nonlinear least-squares regression (i.e. ‘nls’ function in R). We used *a* and *d* as the 10th and 11th traits (*c* was largely invariant and therefore uninformative). Lastly, we estimated pelagic larval duration (PLD), which was the 12th trait. All 12 traits were useful in distinguishing birthdates of settlers and juveniles (electronic supplementary material, figure S1), and most were weakly correlated with one another (electronic supplementary material, table S1).

We trained our 'lda' classification on these 12 traits using settlers and older juveniles (*n* = 702 fish with known birthdates) and a leave-one-out cross-validation (LOOCV). We used this classification alongside an uninformative prior (i.e. unweighted probabilities of assignment) to assign adults to their most likely lunar quarter of birth. We then compared distributions of births across lunar quarters, and across successive life stages: (1) at spawning (i.e. eggs), (2) at settlement, (3) in older post-settlement juveniles, (4a) in females, (4b) in primary males, and (5) in terminal phase males.

### (d) Selection across life-history stages

We quantified the selection on fish born on different lunar quarters by comparing the distributions of births in successive life stages. More specifically, we estimated the pattern of selection (i.e. relative survival during the stage transition) as the relationship between log_10_(*P_j_*_+1,*i*_/*P_j,i_*), and lunar quarter of birth, where *P_j,i_* is the proportion of individuals in stage *j* (i.e. egg, settler, juvenile, female, primary male, terminal phase male) that were born during a given lunar quarter *i* (new, waxing, full or waning moon). Estimates greater than 0 indicate that fish born during that lunar quarter survived relatively well, whereas estimates less than 0 indicate relatively poor survival (i.e. fish born in that lunar quarter were selected against; see [58] for a discussion of indices of selection). We estimated selection patterns for all possible stage transitions.

## 3. Results

### (a) Relationships between spawning patterns and offspring traits

**Figure 1.**
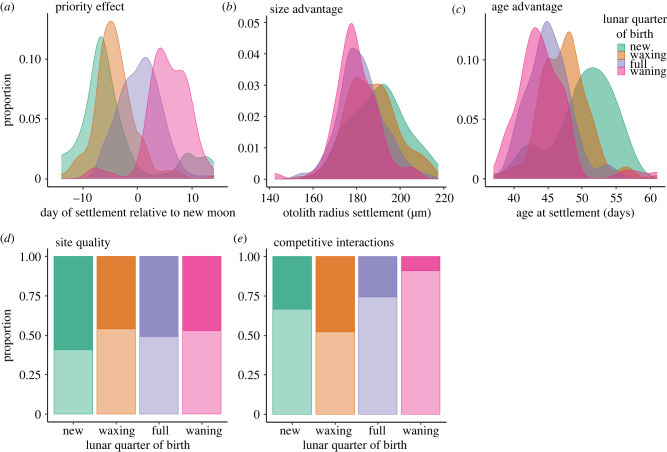
Lunar quarter of birth shapes competitive environments at settlement. (*a*–*c*) Kernel density plots of (*a*) day of settlement relative to the new moon (*x*-values less than 0 indicate settlement before the new moon), (*b*) otolith radius at settlement, and (*c*) age at settlement. (*d*,*e*) Proportions of fish within each lunar quarter of birth that settled (*d*) in offshore (= higher quality, represented by darker shading) versus inshore (= lower quality, represented by lighter shading) sites, and (*e*) into social groups (darker shading) versus alone (lighter shading). Fish born on the new moon were more likely to settle with a priority effect, and at a larger size and age relative to fish born at other times. Fish born on the new moon also were more likely to settle into higher quality (i.e. offshore) sites, and they had a higher probability of settling into social groups relative to fish born during full or waning moons (but not waxing oons).

Lunar quarter of birth shapes the traits and experiences of settling sixbar wrasse (figure 1). Fish that were born at a new moon tended to settle earlier, at a larger size, and at an older age relative to fish born during waxing, full or waning moons (figure 1*a–c*). Fish born at a new moon were also more likely to settle to patch reefs in offshore locations (i.e. higher quality sites; [51,52]). By contrast, fish born at a full moon settled to offshore and inshore locations in similar proportions, and fish born on waxing and waning moons were more likely to settle in inshore locations (i.e. poorer quality sites; figure 1*d*). Most individuals settled to patch reefs as solitary individuals (i.e. no other fish settled to that reef in the same lunar cycle), which likely reduced competition, but also likely reduced the social interactions that trigger maturation to primary male. However, a greater proportion of fish born at the waxing and new moon settled into social groups relative to fish born on the full or waning moon (figure 1*e*). These traits and experiences appear to be a function of lunar quarter of birth, and collectively may set the stage for environmentally determined sex differentiation. Specifically, offspring born at the new moon have traits that increase their likelihood of maturation as a primary male, because they tend to settle into higher quality sites, as part of a social group, and with traits that increase their likelihood of dominance within those social groups (i.e. because they settled earlier, larger and older than others). Experimental evidence from a congener (*Thalassoma bifasciatum*) suggests that such conditions almost always lead to maturation as a primary male [8].

### (b) Trait-based reconstructions of adult birthdates

Residual larval growth histories varied with lunar quarter of birth (figure 2*a*). This striking pattern of variation arises from the effects of moonlight on larval growth, and thus growth patterns are shifted by a fish's birth relative to the lunar cycle [29]. We used the 12 traits associated with this growth pattern to infer lunar quarters of birth for adult fish. Validation based on settlers and juveniles with known birthdates suggested that we could correctly assign individuals to their lunar quarters of birth 50.9% of the time, which was twice that of the random expectation). Misclassifications were almost always to an adjacent lunar quarter of birth, e.g. 87% of all misclassifications (electronic supplementary material, table S2). For settlers and juveniles, the overall distribution of birthdates predicted by the model (i.e. using an LOOCV approach) closely matched the distribution of actual (i.e. known) birthdates for settlers and juveniles (figure 2*b*), providing further validation that this approach may reasonably estimate the distribution of adult birthdates across a lunar cycle.

**Figure 3.**
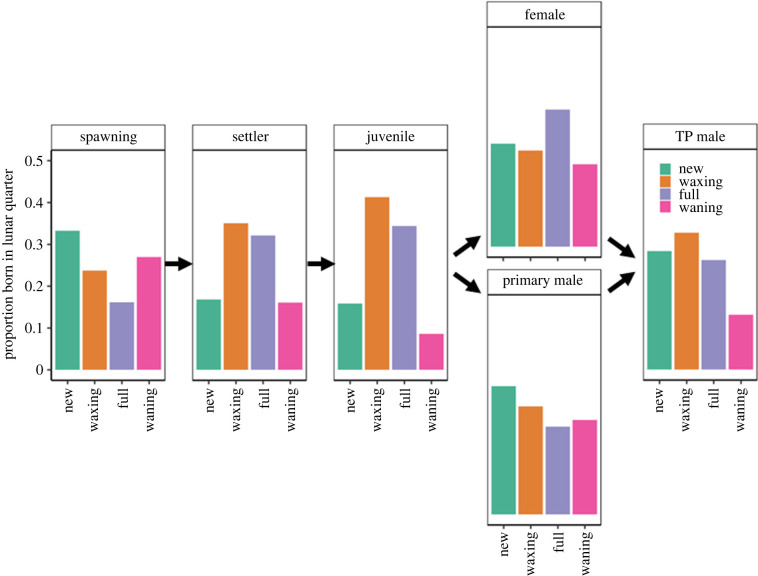
Distribution of births across the lunar cycle vary across developmental stages for the sixbar wrasse. Each panel gives the proportion of sampled fish in a developmental stage born within each quarter of the lunar cycle. Arrows depict sequential stage transitions (and alternative sexual maturation pathways). Proportions of eggs at spawning estimated from [22]. Proportions for settlers (*n* = 411) and juveniles (*n* = 291) estimated from birthdates inferred directly from otolith microstructure. Proportions of females (*n* = 61), primary males (*n* = 62) and terminal phase (TP) males (*n* = 46) estimated from birth quarters inferred from linear discriminant analysis classification. Shifts in the distributions from one stage to another arise from differential survival and/or sexual differentiation.

Trait-based reconstructions of adult birthdates enabled us to evaluate shifts in the distributions of birth patterns across successive life stages (figure 3). While most eggs were spawned at the new and waning moons, most settlers and juveniles had birthdates corresponding to waxing and full moons. However, these patterns were further modified through successive stage transitions. Fish that survived to mature as females were disproportionately born at full moons, whereas fish that survived to mature as primary males exhibited a distribution of births that more closely matched the spawning distribution. Fish that survived to become terminal phase males were a blend of female and primary male distributions: terminal phase males were predominately born during the waxing moon and adjacent moon phases, with relatively few individuals born during a waning moon.

### (c) Selection across life-history stages

**Figure 4.**
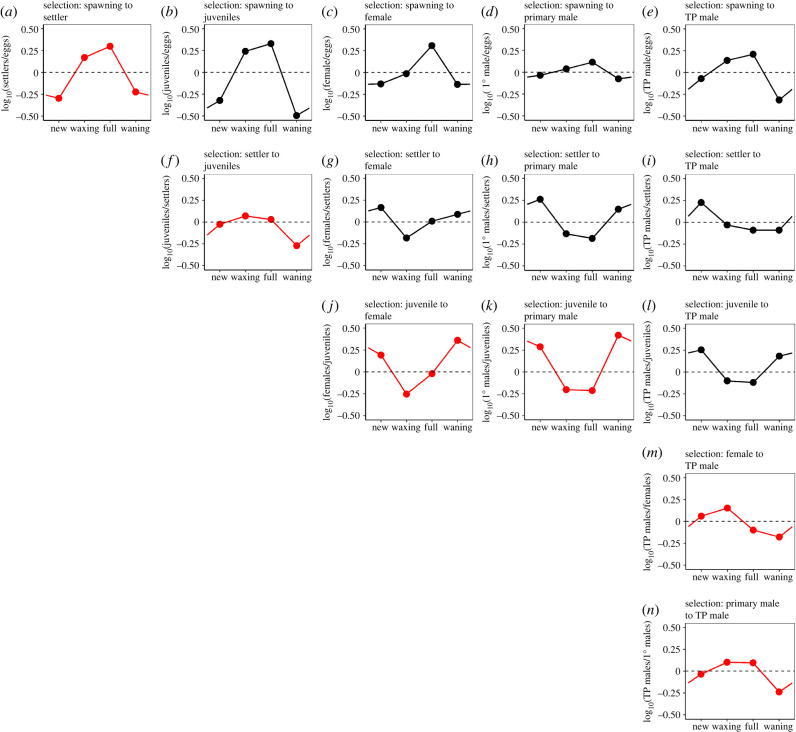
Patterns of selection on lunar quarter of birth. Points indicate relative fitness associated with each lunar quarter of birth, estimated by log ratios of values given in figure 3. Panels depict a matrix of selection patterns for all possible stage transitions (red symbols indicate patterns of selection across sequential stage transitions). TP males, terminal phase males; 1° males, primary males.

The probability of survival varied with lunar quarter of birth, and patterns of selection varied markedly across life stages (figure 4). From spawning to settlement, selection favoured offspring born during the full and waxing moons and acted against offspring born during new and waning moons (figure 4*a*). This pattern of selection was relatively unchanged from settlement through the juvenile stage (cf. figure 4*b*), although offspring born during a waning moon were further selected against (figure 4*f*). Survival from the juvenile stage to sexual maturity counteracted selection in earlier stages by favouring offspring that were born during new and waning moons (figure 4*j*–*l*). Patterns of selection from primary sex differentiation (i.e. female or primary male) to a terminal phase male stage were relatively unmodified, although offspring born during a waning moon were selected against (figure 4*m,n*).

From the parents’ perspective, reproducing during full (and to a lesser extent waxing moons) would maximize production of settlers, juveniles, females and males, while spawning during new and waning moons appears least likely to lead to surviving offspring (figure 4*a*–*e*). Yet spawning is relatively rare during full moons and maximal during new moons (figure 3*a*). This puzzle may be reconciled by (1) selection in later life stages (e.g. figure 4*g*–*l*), and (2) preferential production of males associated with new moon spawning.

## 4. Discussion

Adult sixbar wrasse spawn disproportionately around the new moon, even though spawning at this time results in the death of most offspring [22]. We speculate that this perplexing spawning phenology may be adaptive because it (1) shapes the phenotypes and rearing environments of surviving offspring, and consequently (2) biases their initial maturation to the male phenotype. Collectively, these delayed effects of early experience (i.e. carry-over effects) may compensate for high initial mortality to help explain the overall pattern of adult spawning (i.e. as an evolutionarily stable strategy [59]).

Sixbars are highly iteroparous. Most individuals will spawn multiple times within a lunar month. Parents spawn pelagic eggs, and on a per egg basis, these are relatively energetically inexpensive. Their lunar spawning phenology is evident at the scale of the local population because smaller individuals largely curtail reproduction during the full and waning moons, while larger individuals reproduce more consistently across the lunar month [25]. Spawning at the new moon may be a reproductive tactic for many sixbars, and may be included within their spawning portfolio as part of a diversified bet-hedging approach. More specifically, spawning at the new moon may be a ‘high-risk, high-reward’ investment, akin to buying a lottery ticket for a ‘fitness winner’. Maturing as a primary male may be advantageous in some circumstances, as it affords opportunities to engage in surreptitious matings (via ‘sneaking’ or ‘streaking’ strategies), as well as group spawning events, with many females [25,26]. Additionally, evidence from other *Thalassoma* suggests that primary males have a higher likelihood of attaining a terminal male status (relative to females that change sex) [40], which can further increase an individual's fitness when territorial defence and female monopolization are achievable [27,28]. However, since terminal phase males can arise from either males or females, and because their efficacy is dependent on local population densities [27,28], there may be limited opportunities (or benefits) for parents to influence this outcome.

Spawning at the new moon provides clear advantages to offspring that survive (although most will not). They are more likely to settle earlier, larger and older—all are traits that place them at a competitive advantage. Offspring born at the new moon are also more likely to settle to higher quality habitats, where the deleterious effects of competition are minimized and their post-settlement survival is greatest [51,52]. Lastly, fish born at this time have a relatively greater likelihood of settling into a social group, in which their large size and elevated social status is likely to predispose them to mature as males.

We capitalized on a strong pattern of lunar-cyclic larval growth to infer lunar quarters of birth for adults (because direct estimation of birthdates was not possible). Using this novel approach, we found that offspring that survived to mature as primary males were disproportionately born at new moons. The overall distribution of birthdates for primary males closely resembled the spawning pattern of adults, and consequently, the selection gradient from spawning to primary male was relatively flat (i.e. figure 4*d*). These observations are consistent with our hypothesis that spawning at the new moon may be part of a tactic to make primary males.

We found that fish that initially matured as females were disproportionately born on full moons, suggesting that spawning at this time tends to make females (figure 4*c*). Interestingly, most spawning activity at the full moon is from larger females, whereas smaller females abstain until closer to the new moon [25]. Thus, older females (which have survived longest as females) produce a larger proportion of female offspring, while younger females invest more heavily in a ‘high-risk, high-reward’ strategy that biases maturation of their offspring to primary males. Because we have no information on the relative likelihood of primary males versus females becoming terminal phase males, it is difficult to unravel the tactic that would maximize this outcome (although spawning during the waning moon appears to be ill-advised if this is the goal).

We estimated patterns of selection across all stages in the life history of sixbars and revealed heterogeneous selection pressures that frequently counteracted one another. These complexities may be attributable to stage-specific challenges (e.g. traits may be beneficial in one life stage but a disadvantage in another). Additionally, we speculate that survivors of harsher selective regimes tend to outperform others in future life stages. However, we also note that our approach is not able to determine what proportion of a cohort follows a particular developmental pathway (e.g. the proportion of terminal phase males arising from initial phase males versus females). The complexities revealed in these heterogeneous patterns of selection warrant further attention, and they highlight the general importance of longitudinal studies that consider life histories in their entirety [60].

Phenological patterns are undoubtedly important in the evolution of life-history strategies for many organisms, but information on precise timings of events is often unavailable. Fishes present a unique opportunity to reconstruct the timing of life-history events via their otoliths, but there are limitations to this approach. We were unable to reliably estimate the birthdates of adult sixbars on a lunar calendar owing to the inherent challenges of resolving and counting a large number of daily growth increments without substantial error. For example, a low counting error rate of 1% would equate to an error range of 29 days for an 8-year-old fish, implying that any estimated birthdate within the 29 day lunar cycle would yield no credible information. Hence, we reconstructed birthdates of adults from a set of traits measurable in larval otoliths. Our approach was only able to achieve a 50.9% accuracy, and this is potentially problematic for our other inferences. However, given misclassifications were almost always to an adjacent lunar quarter of birth (i.e. 87% of the cases), and these could go in either direction, we considered the approach to be a reasonably robust estimate of the mean phenological pattern. This belief was reinforced by the close alignment between actual and predicted birth distributions in figure 2*b*. An additional consideration in the interpretation of our results lies with the nature of the samples themselves. Our estimates of spawning, and traits of settlers and juveniles were based on a longitudinal study (i.e. sampling of the same cohorts across their different life stages). Adults were collected over this same period, but their larval experiences would have been many years prior, and these experiences would have spanned many different years. Hence, we make an implicit assumption that the patterns of larval growth in relation to birthdates are consistent across years, and our inferences must be interpreted with appropriate caution.

Delayed effects of prior life experiences (i.e. carry-over effects) appear to be a common feature of marine organisms with complex life cycles [61–67]. Several studies from other systems have documented sex-dependent carry-over effects [60,68,69], and certainly many instances of environmental sex determination [3,4] constitute a form of carry-over effect. Here, we suggest that (1) important carry-over effects may shape post-settlement survival and maturation patterns of the sixbar wrasse, and (2) these carry-over effects may be mediated by the moon, which drives a predictable dynamic of risk and reward in the pelagic rearing environment of many larval fishes and invertebrates [19,22,29]. Collectively, our results suggest the intriguing possibility that adult sixbars (and perhaps other species) may exploit the moon to influence the sex of their offspring.

## Data Availability

All data and code are available from public repositories. Most data used in this paper are available from the Dryad Digital Repository: https://doi.org/10.5061/dryad.zgmsbcc93 [70]. Remaining data and R code for all analyses and figure generation is uploaded as supplementary material [71].
